# Photonic crystal materials and their application in biomedicine

**DOI:** 10.1080/10717544.2017.1321059

**Published:** 2017-05-05

**Authors:** Huadong Chen, Rong Lou, Yanxiao Chen, Lili Chen, Jingya Lu, Qianqian Dong

**Affiliations:** 1 Outpatient Pharmacy and; 2 Clinical Pharmacy, and; 3 Center of Evidence Based Medicine, Affiliated Dongyang Hospital of Wenzhou Medical University, Dongyang 322100, China

**Keywords:** Keywords photonic crystal, biomedicine, drug delivery, biodetection, malignant tumor

## Abstract

Photonic crystal (PC) materials exhibit unique structural colors that originate from their intrinsic photonic band gap. Because of their highly ordered structure and distinct optical characteristics, PC-based biomaterials have advantages in the multiplex detection, biomolecular screening and real-time monitoring of biomolecules. In addition, PCs provide good platforms for drug loading and biomolecule modification, which could be applied to biosensors and biological carriers. A number of methods are now available to fabricate PC materials with variable structure colors, which could be applied in biomedicine. Emphasis is given to the description of various applications of PC materials in biomedicine, including drug delivery, biodetection and tumor screening. We believe that this article will promote greater communication among researchers in the fields of chemistry, material science, biology, medicine and pharmacy.

## Introduction

Recently, biorelated industries have undergone rapid development because of their application in the detection and monitoring of various diseases. Conventional techniques are insufficient because of the complexity of biomolecules and the complicated and high-cost fabrication processes. The detection, delivery and expression of biomolecules such as proteins, DNA and RNA are of vital importance in the prevention and treatment of diseases. Thus a large number of methods and techniques based on nanotechnology have been developed to improve the detection sensitivity for these biomolecules while simultaneously simplifying and reducing the costs of the detection process. These nanomaterials, such as hydrogels, nanoparticles and photonic crystals (PCs), show great promise as components of biosensors (Cui & Lieber, 2001; Alivisatos, 2003; Baksh et al., 2004, Medintz et al., 2005; Fenzl et al., 2014).

Photonic crystals consist of the periodic arrangement of materials with different dielectric constants, which allows them to control and modulate the motion of photons. These materials reflect specific wavelengths of light because of the photonic band gap, which leads them to display distinct structural colors. PC structures are common in nature, such as in opals and butterfly wings, presenting as periodic nanostructures and bright structural colors (Darragh et al., 1966; Srinivasarao, 1999). The structural color of PCs originates from their geometric structure, which is capable of controlling and manipulating the diffraction of light by the periodically arranged structures. After the pioneering work of Yablonovitch and John in 1987 (John, 1987; Yablonovitch, 1987), a large number of PC-related projects was proposed. Over the past several years, PCs have been broadly used in many applications, including sensing (Barry & Wiltzius, 2006; Potyrailo et al., 2007; Huang et al., 2013; Zhang et al., 2015b; Li et al., 2016b), biodetection (Wang et al., 2013; Cai et al., 2014, Massad-Ivanir et al., 2014; Ye et al., 2014; Ayyub & Kofinas, 2015; Cai et al., 2015a,b), displays (Arsenault et al., 2007; Kang et al., 2011; Su et al., 2014), optical devices (Li et al., 2010; Scofield et al., 2011; Li & Meng, 2014; Wan et al., 2016; Aly et al., 2017; Liberal & Engheta, 2017), superconductor (Zhang et al., 2012; Aly et al., 2015; Aly et al., 2016) and solar cells (Agustín et al., 2011; Bayram & Halaoui, 2013; Park et al., 2014).

In recent years, PC materials have attracted more and more attention in the area of biochemistry and biomedicine because of their high sensitivity, selectivity and ability for real-time monitoring (Cunningham & Laing, 2006; Fan et al., 2008). Many new techniques have been developed based on PC materials that have shown obvious advantages in drug delivery, biomolecular detection and tumor screening. This paper reviews the application of PCs in drug delivery, biomedicine and biosensing.

### Basic principles of PC material biosensors

Photonic crystal materials possess periodically modulated dielectric constants and exhibit photonic band gaps, in which electromagnetic wave propagation is forbidden. The photonic band gap can be tuned by external stimulus. Therefore, it is necessary to briefly introduce the physical principles of PC material biosensors. Thus Bragg’s law of diffraction can describe their dynamic diffraction regime:

(1) mλ=2nd×sin

where *m* is the diffraction order, λ is the wavelength of light reflected, *n* is the mean effective refractive index, *d* is the diffracting plane spacing and θ is the angle of incident light.

Based on the light diffraction characteristic of PCs described above, the reflection wavelength of a PC can be altered by changing the distance between particles or the refractive index of the PC film. Therefore, PC materials can be used for sensing applications (Gu et al., 2000; Fenzl et al., 2014; Cai et al., 2015b). A large number of PC-based sensors have been designed to respond to different external stimuli (Ge & Yin, 2011), such as mechanical stress (Foulger et al., 2001; Sumioka et al., 2002), temperature (Espinha et al., 2014; Xu et al., 2015), electric fields (Haurylau et al., 2006; Yuxia et al., 2014), magnetic fields (Ge et al., 2007; Kim et al., 2013) and chemicals (Kim et al., 2014; Wang et al., 2014; Huang et al., 2015).

Generally, the Bragg’s law is the guideline for designing PCs materials, in principle, all the parameters in the Bragg equation contribute to the determination of the photonic band gap, which can be used for the design of PCs. Firstly, the lattice constant is the most widely used parameter to tune the PC optical properties. Under practical conditions, the colloidal particles are usually embedded in polymer matrix, such as hydrogel and elastic polymers, which could be stretched or shrunk to tune the distance of building blocks. Therefore, the corresponding diffraction wavelength and structural color can be modulated by applying various stimuli, such as solvent swelling or deswelling. Secondly, changing refractive index contrast is also an effective method to fabricate responsive PCs. For example, if a stimulus such as biomedicines is introduced to PC systems, the lattice distance and refractive index is increased so that reflection wavelength shift and color change is expected. In addition to changing the parameters in the Bragg diffraction, the periodic structure can also be affected by photonic structures, such as the crystal structures, the relative orientation of the PC and degree of order within the PCs.

In the design of ideal PCs material for biomedicine applications, it usually includes the selection of specific responsive materials, incorporation of the responsive materials into PCs, and optimization of the responsive performance.

### PC materials in drug delivery

Great efforts have been made to explore drug delivery systems, including liposomes (Wang et al., 2010), polymeric micelles (Talelli et al., 2009; Talelli & Hennink, 2011), quantum dots (Muhammad et al., 2011) and silica nanopills (Alba et al., 2015). However, there are still many challenges in maintaining prolonged, steady drug concentrations and improving delivery efficiency. Thus current methods require frequent injections or oral dosing, which is not convenient for patients and can lead to deleterious side effects. PCs are a promising material for use in drug delivery systems because of their biosafety and tunable optical characteristics, which allow them to be loaded with a large number of agents and to deliver drugs in a controllable manner, leading to higher drug delivery efficiency and lower side effects. In addition, the ability to easily modify the surface of PCs is beneficial for coupling them to biomolecules and extends their applicability within the realm of biomedical sciences (Biju, 2014).

To enhance drug loading and release capabilities, inverse opals (IOs) with three-dimensional periodic macroporous structures and large surface areas are desirable materials. The colloidal crystal template technique(Stein & Schroden, 2001, Jiang et al., 1999) is an effective way to fabricate three-dimensional IOs. As for drug delivery materials, IO materials are usually composed of nontoxic and biocompatible polymers and nanoparticles; the polymers lead to great biocompatibility and the macropores of the particles provide channels for drug loading. Furthermore, if appropriate polymer matrixes are chosen, the fabricated PCs can be immediately responsive to specific environmental stimuli, leading to the shrinking or swelling of the scaffold and subsequently a shift in reflective wavelength. Thus, PCs can be used not only to tune the delivery of a drug but also to act as self-monitoring sensors, both of which have great potential in drug delivery systems.

**Figure 1.**
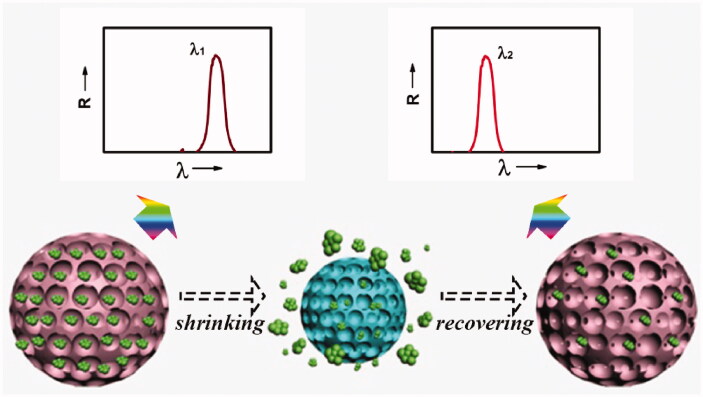
Overview of the pNIPAM hydrogel IO. Schematic of the self-reporting feature of the pNIPAM hydrogel IO particles during drug release (green circles). The accompanying blue shift in the reflection peak of a particle at triggered release is indicated by the two wavelengths λ1 and λ2 (Zhang et al., 2015a).

Gu et al. reported the fabrication of poly (*N*-isopropylacrylamide) (pNIPAM) hydrogel IO scaffolds loaded with dextran and the calcium alginate hydrogel, which could be applied to particle-based drug delivery (Zhang et al., 2015a) (Figure 1). As is typically carried out, the hydrogel was prepared by removing the template of silica colloidal crystal beads. As the pNIPAM hydrogel IO scaffolds shrank and swelled at different temperatures, this could make it possible to control the release of drugs by modulating the environmental temperature. In addition, the reflection wavelength of the hydrogel IO would blue shift when the drug was released. Therefore, the process of drug delivery was effectively detected in real time. Such characteristics make IO particles ideal materials for drug delivery systems.

Electrically responsive colloidal crystal films can also be applied to drug delivery systems. Svirskis et al. prepared electrically responsive three-dimensional ordered macroporous polypyrrole (PPy) IO films with the template method and then used these films for triggered dexamethasone release (Seyfoddin et al., 2015). Corticosteroids such as dexamethasone are frequently used to treat posterior uveitis and could be used to eliminate this inflammatory condition, although their ocular bioavailability is limited and frequent injections are required (Roth et al., 2003; Bansal et al., 2012). Dexamethasone phosphate (DexP) was incorporated into the PPy IO film during fabrication, and the resulting PPy IO–DexP film had a large surface area and three-dimensionally ordered macroporous structure, which led to great electrical responsiveness. Compared with conventional non-porous films, the PPy IO films had a larger amount of drug release. The electrically responsive PPy IO films also showed enhanced drug loading and releasing capabilities for risperidone, as compared to non-porous PPy films (Xu et al., 2008; Sharma et al., 2013).

Such stimuli-responsive PC-based drug delivery systems have broad application as drug delivery implants, as they allow adjustment of the drug release based on an electrical or temperature stimulus according to the needs of the patient and provide monitoring of drug release in real time. These features make functional PC materials promising for implantable devices, which could replace conventional treatments and enhance the safety and efficacy of drugs. Thus, PC-based drug delivery systems have great potential for clinical applications.

### PC materials in biodetection

Biosensors are analytical devices composed of biologically responsive materials and signal transducers that convert a biological response into electrochemical, optical, electrical or magnetic signals. Among these, optical biosensors have important roles in biomedical research, healthcare, pharmaceuticals, environmental monitoring and homeland security because of their ability to perform remote sensing and provide vital and complementary information (Fan et al., 2008). PC-based biosensors provide new methods to simplify the read-out and lower the cost of bioassays, which makes these biosensors more versatile and widely used today (Heeres et al., 2009; Hu et al., 2009; Wang et al., 2013; Lu et al., 2014; Ye et al., 2014; Moirangthem et al., 2016; Rong et al., 2016). Typically, biosensors make use of biological components such as antibodies, aptamers, enzymes and gene probes to recognize a specific target. Such an approach, for example, allows the determination of creatinine concentration in bodily fluids, providing an important diagnostic tool for renal dysfunction (Soldatkin et al., 2002). Asher et al. proposed a new PC motif for the detection and quantification of creatinine for which the detection limit is 6 μM (Sharma et al., 2004). The PC materials for this detector are composed of polystyrene crystalline colloidal arrays and polyacrylamide hydrogels. To realize quantitative detection, a pH-responsive polymerized crystalline colloidal array (PCCA) was combined with an enzyme reaction that liberates OH^–^. In this series of reactions, creatinine is first hydrolyzed by creatinine deaminase, which leads to the release of hydroxide ions. Then 2-nitrophenol in the hydrogel titrates the resulting change in pH, which causes the PCCA hydrogel to swell. The creatinine concentration is then visualized and quantified by measuring the diffraction red shift.

**Figure 2.**
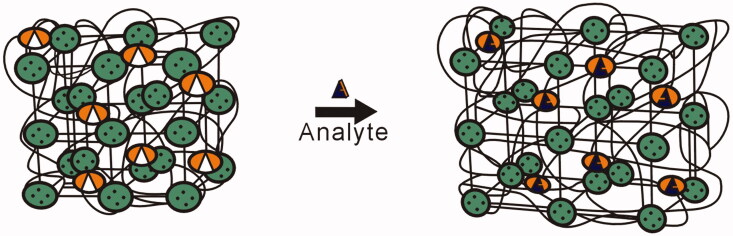
PC sensing materials that consist of CCA surrounded by a polymer hydrogel. In this example, the hydrogel volume swells in response to the interaction between glucose and the molecular recognition element (Alexeev et al., 2005).

PCs can also be responsive to glucose, which offers a new method for diagnosing diabetes (Alexeev et al., 2003; Nakayama et al., 2003; Muscatello et al., 2009). The ability to diagnose diabetes is one of the most successful applications of PC biosensors to date. Takeoka and his coworkers designed a colorimetric sensor for detecting glucose levels in blood (Nakayama et al., 2003). The glucose-sensitive IO gel was prepared by using a phenylboronic acid derivative as the sensing monomer. The additional glucose complexes with phenylboronic acid and increases the volume of the hydrogel, further changing the reflection peak and structural color of the gel (Figure 2). Asher et al. have subsequently improved upon this method by using a new sensor material that can be used to sense glucose at low concentrations in tear fluid (Alexeev et al., 2005). This high-sensitivity glucose-sensing material could be incorporated into contact lenses or ocular inserts to detect glucose concentration and may provide greater convenience for patients and improved glycemic control.

As another example of a PC-based biosensor, cholesterol can be detected by attaching the cholesterol oxidase (ChOx) to an epoxide-functionalized PCCA (Maurer et al., 2008). Compared with the unbound enzyme, the bound cholesterol oxidase retains 85% of its initial activity. When the PCCA was exposed to 5.0 mM cholesterol, the diffraction wavelength was red-shifted ∼60 nm. Therefore, the material is a promising optical cholesterol sensor. Gu et al. reported the synthesis of three-dimensionally structured PC carbon inverse opal rods (CIORs) by using the template method, which were then used for nonenzymatic cholesterol detection (Zhong et al., 2015). The porous CIORs are permeable to cholesterol but can block out blood cells and proteins that are larger than the pore size, which makes it possible for the detection of cholesterol in human serum and blood. The prepared CIORs have low fabrication costs, are simple to use and have a fast reaction time, features that make them suitable for clinical diagnosis.

### PC materials for screening malignant tumors

Malignant tumors are a leading cause of death worldwide (Jemal et al., 2011), and early diagnosis and treatment is of vital importance to reduce the mortality of affected individuals (Creeden et al., 2011). Tumor markers in the serum can offer effective information for the screening, diagnosis and prognosis of malignant tumors (Joerger & Huober, 2012; Kelloff & Sigman, 2012). Correspondingly, a large number of methods and related machines have been developed to detect tumor markers, such as chemiluminescence immunoassay (CLIA), rate nephelometry and so on (Ge et al., 2012). Alpha-fetoprotein (AFP) and carcinoembryonic antigen (CEA) are clinically important tumor markers. Silver nanoshell silica PC beads (Ag-SPCBs) have been synthesized to be used for tumor detection (Li et al., 2016a). The resultant Ag-SPCBs not only show a highly uniform structure but also have tremendous surface enhanced raman scattering (SERS) signal enhancement. When Ag-SPCBs were immobilized by two different antibody molecules, AFP and CEA could be determined in one test tube. The detection limits of CEA and AFP were 6.6 × 10^−6^ and 7.2 × 10^−5 ^ng mL^−1^, respectively, with the one-tube Ag-SPCB method, which is much lower than the results of previously reported SERS assay methods (Chon et al., 2009**)**. PC materials could also be used for multiple electrochemiluminescent immunoassays for tumor marker detection, which could be further developed for practical clinical detection of serum CEA and AFP levels (Pei et al., 2010).

During chemotherapy, the generation of drug resistance, especially multidrug resistance, in tumor cells leads to the failure of this treatment option. As multidrug resistance is generated, there is enhanced expression of multidrug resistance transporters on the surface of malignant tumor cells (Fojo et al., 1986; Higgins, 2007). Therefore, multiplex detection of genes that encode proteins such as multidrug resistance 1 (MDR1) and multidrug resistance-associated protein 1 (MRP1) is beneficial for the prognosis of patients. An easily carried out method based on silica colloidal crystal beads (SCCBs) has been developed for the multiplex analysis of such genes (Yang et al., 2012). The conventional fluorescent dyes encoding is easily quenched under light and heat, leading to the loss of encoding. However, the PC-based encoding is a physical encoding strategy, which could be used as micro-carrier of encode proteins with stable encoding. In addition, the porous structure of the bead array results in a high surface-to-volume ratio, which greatly improves the sensitivity of detection relative to non-porous structures. The detection limit of this method could be as low as 10^−19 ^ M and should allow the simultaneous analysis of the expression of multiple genes.

## Conclusions

This review has described the use of PC materials for biomedical applications, including drug delivery, biodetection and tumor screening. PC-based biosensors show great promise for clinical detection because of their low cost, easy read-out and real-time monitoring features. For example, PC-based glucose detection techniques are relatively well established and have even been commercialized. Compared to the conventional methods, visibly detectable color change is achieved by applying a biochemical stimulus, which potentially have a large market. However, other techniques such as multiplex detection and gene screening are still in their infancy and some challenges need to be addressed. (1) Major effort is to develop self-assembly approaches to meet the requirements for manufacturing. Fabrication of large scale and high quality photonic structures are required in the transition from laboratory to industrial practice, including further improve their resolution and discrimination. (2) Secondly, most of the biosensors produce single-shot measurements only and are not able to monitor a species continuously, which is typically required for many types of clinical diagnosis. For example, the detection of glucose is single-use activity, dynamic monitoring is not achieved. (3) Thirdly, the selectivity of PC sensors also needs to be improved. The PC-based sensors, such as creatinine, are often affected by the ionic strength of a solution or by other analytes. When designing new systems with improved performance, we should learn from the natural photonic structures, which appears to have excellent properties and provides replicated bio-templates for the design of novel photonic materials.

It could be found that the PC-based materials for biomedicine application have attracted considerable attention in a short period of time. This is not only because of the unique photonic properties of PC materials but also benefits from the combining of chemistry and biology, which provides a good direction for developing PC materials. We believe that PC materials will have a bright future in biomedicine and clinical applications due to their unique optical properties.
